# Data for a proteomic analysis of p53-independent induction of apoptosis by bortezomib

**DOI:** 10.1016/j.dib.2014.09.003

**Published:** 2014-10-19

**Authors:** Azmi Yerlikaya, Emrah Okur, Ahmet Tarık Baykal, Ceyda Acılan, İhsan Boyacı, Engin Ulukaya

**Affiliations:** a Dumlupınar University, Faculty of Medicine, Department of Medical Biology, Kütahya, Turkey; b Dumlupınar University, Art and Science Faculty, Department of Biology, Kütahya, Turkey; c İstanbul Medipol University, Medical School, Department of Medical Biochemistry, İstanbul, Turkey; d TÜBİTAK, MAM, Genetic Engineering and Biotechnology Department, Gebze, Kocaeli, Turkey; e İstanbul Medipol University, Vatan Clinic, İstanbul, 34214, Turkey; f Department of Medical Biochemistry, Uludağ University, Bursa, Turkey

## Abstract

This data article contains data related to the research article entitled, “A proteomic analysis of p53-independent induction of apoptosis by bortezomib in 4T1 breast cancer cell line” by Yerlikaya et al. [1]. The research article presented 2-DE and nLC-MS/MS based proteomic analysis of proteasome inhibitor bortezomib-induced changes in the expression of cellular proteins. The report showed that GRP78 and TCEB2 were over-expressed in response to treatment with bortezomib for 24 h. In addition, the report demonstrated that Hsp70, the 26S proteasome non-ATPase regulatory subunit 14 and sequestosome 1 were increased at least 2 fold in p53-deficient 4T1 cells. The data here show for the first time the increased expressions of Card10, Dffb, Traf3 and Trp53bp2 in response to inhibition of the 26S proteasome. The information presented here also shows that both Traf1 and Xiap (a member of IAPs) are also downregulated simultaneously upon proteasomal inhibition. The increases in the level of Card10 and Trp53bp2 proteins were verified by Western blot analysis in response to varying concentrations of bortezomib for 24 h.

## Specifications table

**Table:** 

Subject area	Medicine, Biology
More specific subject area	Molecular Biology, Cancer Biology
Type of data	Table, figure
How data was acquired	Real-Time PCR, Western blotting system
Data format	Analyzed
Experimental factors	p53-null mouse 4T1 cell line were treated with isotonic solution or varying doses of bortezomib.
Experimental features	*Total RNA is isolated and then converted to cDNA. The cDNA samples* quantified with Roche Light Cycler 480 platform. The Western blots were carried out with Amersham ECL Western blotting kit.
Data source location	Kütahya, Turkey
Data accessibility	The data are supplied with this article.

## The value of the data

• Proteasome inhibition regulates the expression of Card10, Dffb, Traf3, Trp53bp2, Bcl2-like 1, Fadd, Traf1 and Xiap proteins in p53-*null* 4T1 breast carcinoma cells.
• These proteins have critical roles in cellular homeostasis and cancer cell survival [2,3,4].
• The data are useful for understanding the role of proteasome in cancer development.
• The data are also valuable for elucidating the mechanism of regulation of these genes under the conditions of proteasomal inhibition.

## 1 Data, experimental design, materials and methods

The data shown here report the changes in the expression levels of apoptosis-related genes in p53-*null* 4T1 mouse breast carcinoma cell lines [5,6]. The changes in the expression of apoptosis-related genes were examined by real-time PCR in response to 100 nM bortezomib (also known as Velcade^TM^ or PS-341) for 24 h. Eleven apoptosis-related genes were upregulated in response to 100 nM bortezomib-treatment. Additionally, Bcl2l1, Fadd, Traf1 and Xiap genes are found to be downregulated in a p53-independent manner in 4T1 cells.

*Real-Time PCR* – Mouse 4T1 cell lines were seeded in 60×15 mm^2^ petri dishes and treated with 100 nM bortezomib at the logarithmic phase of the growth for 24 h. Afterwards, RNA was isolated using RNeasy mini kit and QIAcube instrument according to the manufacturer׳s protocol (SABioscience, Frederick, MD, USA). Genomic DNA elimination was performed using the same amount of total RNA (1 μg from each sample), and then cDNA was synthesized for each sample using RT^2^ First Strand kit (SABioscience, Frederick, MD, USA). Equal amounts of cDNAs from control and treated samples were mixed with RT^2^ SYBR Green Mastermix and 25 μl PCR component mix was added to each well of mouse RT^2^ profiler apoptosis PCR array using a 12-channel pipettor. After tightly sealing the RT^2^ PCR array with optical adhesive film, the absolute quantification was performed with Roche Light Cycler 480 platform using 1 cycle of hotstart (10 min at 95 °C) and 45 cycles of amplification (15 s at 95 °C and 1 min at 60 °C). Data were normalized using housekeeping genes (beta-actin, beta-2 microglobulin, glyceraldehyde-3-phosphate dehydrogenase and beta-glucuronidase) and analyzed by comparing 2–Δ*C_T_*.

The data presented here shows that inhibition of the 26S proteasome causes significant changes in the expression of Card10, Dffb, Traf3 and Trp53bp2 genes. In addition, four genes were found consistently downregulated in response to treatment with bortezomib for 24 h. The genes downregulated were Bcl2-like 1, Fas (TNFRSF6)-associated via death domain, Tnf receptor-associated factor 1 and X-linked inhibitor of apoptosis proteins (Table 1).

*Western blotting* – The ECL Western blotting kit was used according to manufacturer procedure (GE Healthcare, Stockholm, Sweden). A total of 50 μg protein from each sample was separated on a 12% SDS-PAGE. Afterwards, proteins were transferred to PVDF membranes at 70 V for 2 h. After the transfer, the PVDF membranes were washed briefly with methanol and left for drying for 15 min to enhance the protein binding. The PVDF membranes were again reactivated by methanol. The membranes were blocked by 5% non-fat dried milk in TBS-T. The membranes were then incubated with anti-Card10 (1:500) and anti-Trp53BP2 (1:500) for 1 h. For loading control, the membranes were probed with anti-β-actin antibody (1:5000) in TBS-T for 1 h. The membranes were then incubated with HRP-conjugated anti-rabbit secondary antibody (1:5000 dilution) in TBS-T for 1 h.

**Fig. 1:**
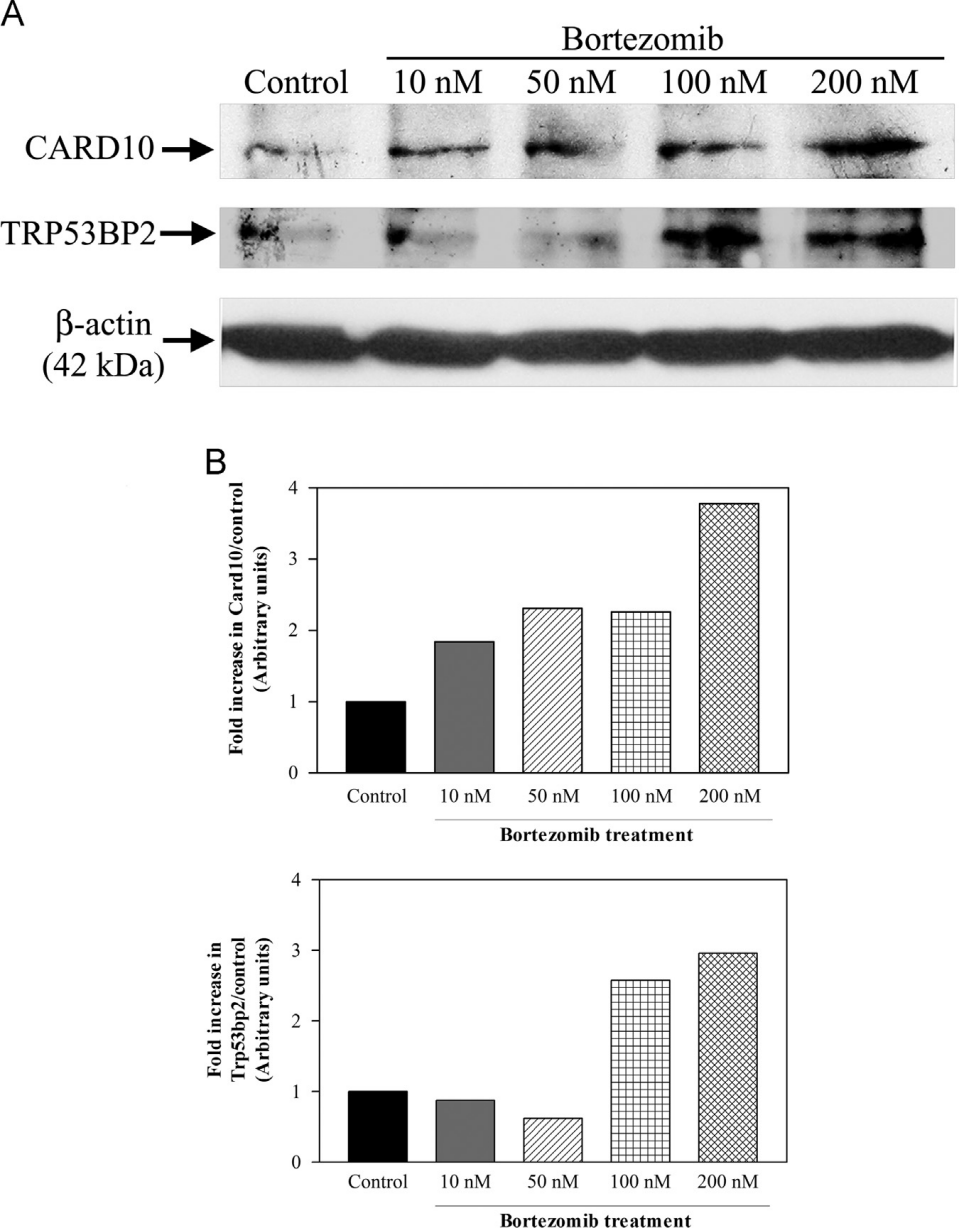
(A) Examination of Card10 and Trp53bp2 proteins by Western blotting. 4T1 cells were treated with various concentrations of bortezomib (10, 50, 100 and 200 nM) for 24 h. And then the levels of Card10 (upper panel) and Trp53bp2 (middle panel) were analyzed by rabbit anti-Card10 (1:500 dilution) or mouse anti-Trp53bp2 (1:500 dilution) antibodies, respectively. The lower panel shows the level of loading control β-actin. The result is a representative of 2 independent experiments, each run in duplicates. (B) Quantitation of Card10 and Trp53bp2 protein bands seen in Panel A by GelQuantNET. The data are graphed by Graphpad Prism 3.03 program. The control level is set to 1.

The Western blot analysis results indicated that the increases in Card10 (Fig. 1A, upper panel) and Trp53bp2 (Fig. 1A, middle panel) proteins were corroborated in response to proteasomal inhibition by various concentrations of bortezomib for 24 h. The examination of β-actin level (Fig. 1A, lower panel) showed that the changes in the protein levels of Card10 and Trp53bp2 were not simply due to higher protein loading. As can be seen in Fig. 1B, when the cells were treated with different doses of bortezomib, a threshold-dependent increase in Card10 protein was clearly observed. With 10 nM, 50 nM, 100 nM and 200 nM, 1.84 fold, 2.31 fold, 2.26 fold and 3.78 fold increases were detected, respectively. On the other hand, the increase in the level of Trp53bp2 protein was observed with only higher doses of bortezomib (i.e., 100 nM and 200 nM) (Fig. 1B).

## Figures and Tables

**Table 1:** Real-time PCR measurements. Mouse 4T1 cells were seeded in 60×15 mm^2^ dishes and treated with 100 nM bortezomib for 24 h, followed by RNA isolation using RNeasy mini kit and QIAcube instrument. The quantitative PCR measurements were performed using RT^2^ SYBR Green Mastermix and Roche Light Cylcer 480 platform. The average Δ*C*_*T*_ values and the fold changes of genes upregulated or downregulated are indicated in the table. The data are the average of two independent experiments.

**Gene Symbol**	**Description**	**Avg Δ*****C***_***T***_	**Fold change**	
			Control	Bortezomib

**Upregulated genes**				
**Atf5**	Activating transcription factor 5	7.1075	5.8725	2.3538
**Bag3**	Bcl2-associated athanogene 3	4.9875	3.0025	3.9586
**Bcl2a1a**	B-cell leukemia/lymphoma 2 related protein A1a	16.4775	14.2725	4.6107
**Card10**	Caspase recruitment domain family, member 10	4.1475	1.7425	5.2964
**Cd40**	CD40 antigen	9.8975	8.1525	3.3519
**Dffb**	DNA fragmentation factor, beta subunit	10.0775	8.5125	2.9588
**Gadd45a**	Growth arrest and DNA-damage-inducible 45 alpha	5.0075	3.5225	2.7992
**Tnfrsf10b**	Tumor necrosis factor receptor superfamily, member 10b	4.8875	3.7625	2.181
**Traf3**	Tnf receptor-associated factor 3	5.7975	4.5725	2.3376
**Trp53bp2**	Transformation related protein 53 binding protein 2	5.8075	4.7425	2.0922
**Hsp90ab1**	Heat shock protein 90 alpha (cytosolic), class B member 1	−1.2725	−2.7675	2.8186

**Downregulated genes**				
**Bcl2l1**	Bcl2-like 1	4.0875	5.4325	0.3937
**Fadd**	Fas (TNFRSF6)-associated via death domain	6.8175	7.9925	0.4429
**Traf1**	Tnf receptor-associated factor 1	5.3375	6.7425	0.3776
**Xiap**	X-linked inhibitor of apoptosis	5.9275	7.0425	0.4617
